# Gene Expression Profiling of Ampullary Carcinomas Classifies Ampullary Carcinomas into Biliary-Like and Intestinal-Like Subtypes That Are Prognostic of Outcome

**DOI:** 10.1371/journal.pone.0065144

**Published:** 2013-06-11

**Authors:** Michael J. Overman, Jiexin Zhang, Scott Kopetz, Michael Davies, Jiang Zhi-Qin, Katherine Stemke-Hale, Petra Rümmele, Christian Pilarsky, Robert Grützmann, Stanley Hamilton, Rosa Hwang, James L. Abbruzzese, Gauri Varadhachary, Bradley Broom, Huamin Wang

**Affiliations:** 1 Department of Gastrointestinal Medical Oncology, The University of Texas M. D. Anderson Cancer Center, Houston, Texas, United States of America; 2 Department of Biostatistics, The University of Texas M. D. Anderson Cancer Center, Houston, Texas, United States of America; 3 Department of Pathology, The University of Texas M. D. Anderson Cancer Center, Houston, Texas, United States of America; 4 Department of Melanoma, The University of Texas M. D. Anderson Cancer Center, Houston, Texas, United States of America; 5 Department of Systems Biology, The University of Texas M. D. Anderson Cancer Center, Houston, Texas, United States of America; 6 Department of Surgical Oncology, The University of Texas M. D. Anderson Cancer Center, Houston, Texas, United States of America; 7 Institute of Pathology, University of Regensburg, Regensburg, Germany; 8 Department of Visceral, Thoracic, and Vascular Surgery, University Hospital Carl Gustav Carus, Dresden, Germany; University of Texas MD Anderson Cancer Center, United States of America

## Abstract

**Background:**

Adenocarcinomas of the ampulla of Vater are classified as biliary cancers, though the exact epithelium of origin for these cancers is not known. We sought to molecularly classify ampullary adenocarcinomas in comparison to known adenocarcinomas of the pancreas, bile duct, and duodenum by gene expression analysis.

**Methods:**

We analyzed 32 fresh-frozen resected, untreated periampullary adenocarcinomas (8 pancreatic, 2 extrahepatic biliary, 8 duodenal, and 14 ampullary) using the Affymetrix U133 Plus 2.0 genome array. Unsupervised and supervised hierarchical clustering identified two subtypes of ampullary carcinomas that were molecularly and histologically characterized.

**Results:**

Hierarchical clustering of periampullary carcinomas segregated ampullary carcinomas into two subgroups, which were distinctly different from pancreatic carcinomas. Non-pancreatic periampullary adenocarcinomas were segregated into two subgroups with differing prognoses: 5 year RFS (77% vs. 0%, p = 0.007) and 5 year OS (100% vs. 35%, p = 0.005). Unsupervised clustering analysis of the 14 ampullary samples also identified two subgroups: a good prognosis intestinal-like subgroup and a poor prognosis biliary-like subgroup with 5 year OS of 70% vs. 28%, P = 0.09. Expression of CK7+/CK20- but not CDX-2 correlated with these two subgroups. Activation of the AKT and MAPK pathways were both increased in the poor prognostic biliary-like subgroup. In an independent 80 patient ampullary validation dataset only histological subtype (intestinal vs. pancreaticobiliary) was significantly associated with OS in both univariate (p = 0.006) and multivariate analysis (P = 0.04).

**Conclusions:**

Gene expression analysis discriminated pancreatic adenocarcinomas from other periampullary carcinomas and identified two prognostically relevant subgroups of ampullary adenocarcinomas. Histological subtype was an independent prognostic factor in ampullary adenocarcinomas.

## Introduction

Ampullary adenocarcinomas are cancers that are anatomically centered at the ampulla of Vater. Though classified by the World Health Organization as cancers of the extrahepatic bile duct, ampullary adenocarcinomas have better prognosis when compared to similarly staged pancreatic or biliary adenocarcinomas. [1]–[3] Three distinct epithelial linings (duodenal, biliary, and pancreatic) converge at the ampulla of Vater, with pancreatic and biliary epithelium merging within the ampulla of Vater to form a true ampullary epithelium. Thus, it is uncertain whether adenocarcinomas originating at the ampulla of Vater represent a homogenous carcinoma group reflective of a true ampullary epithelium or a heterogeneous group reflective of these various epithelial origins.

Given the uncertain epithelial origin of ampullary adenocarcinomas, a number of studies have attempted to identify prognostically differing subtypes. The first approach to subtype ampullary adenocarcinomas was based upon segregating cases by histology as either pancreaticobiliary type or intestinal type. [4] Though a number of studies have found this approach to have statistically significant prognostic impact [5]–[8], other studies have not [9]–[11]. More recently studies have investigated additional markers such cytokeratin expression, mucin expression, microsatellite instability, and intestinal-specific markers to identify prognostically distinct subgroups of ampullary adenocarcinomas. [5], [7], [10]–[16] For example, expression of the intestinal markers, CDX-2 and CDX-1, were recently shown to correlate with improved OS in a cohort of 53 patients [13], but this finding was not validated in subsequent studies [5], [12]. Though these studies taken together have been suggestive of heterogeneity within ampullary adenocarcinomas, interpretation of these results has been limited by small sample size and variability in classification methodology. Thus, at present, no single method has consistently identified prognostically relevant subgroups of ampullary adenocarcinomas.

In order to improve the understanding of the heterogeneity within ampullary adenocarcinomas, we sought to classify ampullary adenocarcinomas at a molecular level by comparing the mRNA gene expression from clinically-annotated specimens of ampullary adenocarcinomas to the expression patterns of pancreatic, duodenal, and biliary adenocarcinomas. In addition transcriptional profiles were compared to patient characteristics and clinical outcomes. The patterns of the expression and activation of proteins in signaling networks were also assessed using reverse phase protein arrays (RPPA). This study shows a molecular distinction between ampullary and pancreatic adenocarcinomas, identifies robust prognostic subgroups of ampullary adenocarcinomas, and implicates a number of targetable signaling pathways in the pathogenesis of these tumors.

## Methods

### Periampullary Adenocarcinoma Study Population

Fifty-two treatment naïve periampullary adenocarcinoma samples from pancreaticoduodenectomies were identified from The University of Texas MD Anderson Cancer Center (UTMDACC) frozen tumor bank from 2002 to 2009. The specific tumor site of origin for each sample (ampullary, duodenal, pancreatic, or extrahepatic biliary) was based upon the original pancreaticoduodenectomy pathology report. The cases in which a specific tumor site of origin could not be determined from the surgical resection specimen were not included. Tumor samples represented grossly dissected snap-frozen tumors (10 to 25 mg) stored in liquid nitrogen. A hematoxylin & eosin (H & E) stained section from each frozen tumor sample was prepared and reviewed by a gastrointestinal pathologist (HW) for histological verification and for determination of both histological grade and ampullary histological subtype. The final study population represented 32 samples that remained after the application of the following exclusion criteria: no histologically verified adenocarcinoma present (n = 5), ≤70% tumor cells (n = 4), and inadequate RNA quality with a RNA integrity number ≤4.5 (n = 11). All studies were performed under a UTMDACC institutional review board approved protocol.

### Gene Expression Profiling and Analysis

Total RNA was extracted using the TRIzol method (Invitrogen, Carlsbad, CA). RNA was purified using the RNeasy mini-kit (Qiagen, Alameda, CA) and the quality of RNA was assessed on an Agilent 2100 Bioanalyzer and quantified using Nano Drop ND-1000 (Nano Drop, Wilmington, DE). Samples were amplified, labeled, and 10µg of cRNA was hybridized to the HG-U133 Plus 2.0 Affymetrix GeneChip array according the manufacturer’s protocol (Affymetrix, Santa Clara, CA). RMA (Robust Multichip Average) expression values were calculated from the microarray data and the hierarchical clustering was performed using ward linkage and Pearson correlation distance with probsets that were called present on at least 3 arrays. [17] One-way ANOVA was used to identify genes that are differentially expressed in at least one tissue type. The p-values from one-way ANOVA were modeled using a beta-uniform mixture (BUM) model. With the use of a false discovery rate (FDR) of 0.05, we then identified 1353 such differentially expressed genes (DEGs). The Tukey Honestly Significant Difference test, in conjunction with one-way ANOVA results, identified 133 significantly DEGs between pancreatic and duodenal adenocarcinomas (P<0.01). All samples underwent unsupervised hierarchical clustering and supervised clustering based upon DEGs between pancreatic and duodenal cases. Differences between clusters was assessed using Statistical Significance of Clustering (SigClust). In addition unsupervised hierarchical clustering of only ampullary samples was done with probsets that were called present on at least 1 ampullary array. The data discussed in this publication have been deposited in Gene Expression Omnibus accession number GSE39409 (http://www.ncbi.nlm.nih.gov/geo/query/acc.cgi?acc=GSE39409) and an auto executable Sweave file regarding the preformed analyses is available upon request.

### Molecular and Histopathological Analysis

The corresponding formalin-fixed paraffin embedded tissue from each frozen tumor sample was identified and 5-µm unstained slides of both representative tumor and normal small bowel mucosa were created. Immunohistochemical staining for CDX-2, cytokeratin-7 (CK-7), and CK-20 was conducted and considered positive if 10% or more of the tumor cells exhibited immunoreactivity. For microsatellite instability (MSI) analysis, DNA samples were isolated from each frozen tumor sample and its matched normal control tissue, which was macro-dissected from the paraffin embedded tissue. Samples with MSI at two or more loci were classified as MSI-high. Activating mutations in KRAS, BRAF, and PIK3CA mutations were identified using the Sequenom® high-throughput MassARRAY platform (Methods S1).

### Reverse Phase Protein Array Analysis

Quantitative assessment of protein expression for 140 validated proteins was conducted using reverse phase protein array (RPPA) technology at the MD Anderson Functional Proteomics Core Facility. [18], [19] Uniform protein concentrations were created and a logarithmic value was generated, which reflected the relative amount of each protein in each sample. RPPA data for the ampullary samples was independently normalized and median-centered. Fisher’s exact T-test was used to compare mean values and unsupervised hierarchical clustering using Cluster 3.0 and Treeview (Standford University) was performed on the differentially expressed proteins (P≤0.05).

### Ampullary Adenocarcinoma Validation Population

We searched the UTMDCC tumor registry and pathology database to identify paraffin embedded archival tumor tissue from a separate cohort of 86 treatment naive ampullary adenocarcinomas who underwent a pancreaticoduodenectomy. Upon review of the original pathological diagnosis, 6 cases were found to be termed periampullary carcinomas and were excluded. The determination of histological subtypes (intestinal, pancreaticobiliary, or mixed) was based upon review of the full resection specimen from the Department of Pathology by a gastrointestinal pathologist (HW). For determination of CDX-2, CK7, and CK20 immunoreactivity, a tissue microarray was constructed and stained (Methods S1).

### Survival Analysis

Association between categorical clinical variables was determined by the Fisher’s exact test. Survival curves were generated using the Kaplan-Meier method and survival differences were determined with the log-rank test. The univariate Cox proportional hazards regression model for overall survival (OS) and relapse-free survival (RFS) tested age, histological grade, histological subtype, tumor stage, resection margin status, lymph node involvement, adjuvant treatment, and immunohistochemical variables. Cox proportional hazards models were fitted for multivariate analysis. After interactions between variables were examined, a backward stepwise procedure was used to derive the best-fitting model. Statistical analyses were performed using Stata MP version 10.1 and R version 2.12.0.

## Results

### Periampullary Adenocarcinoma Gene Expression Profiling

**Figure 1:**
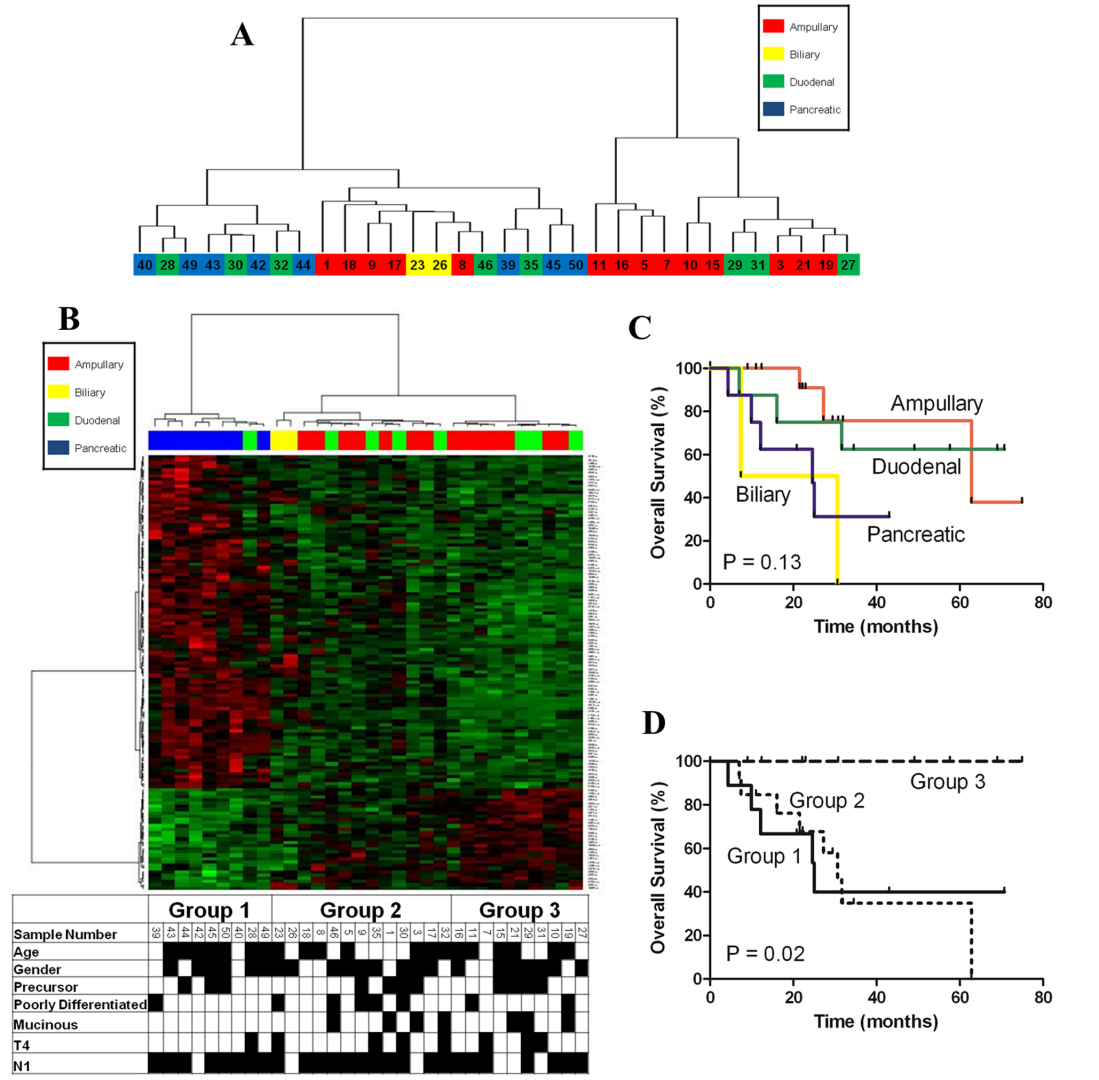
Unsupervised hierarchical clustering of all 32 periampullary adenocarcinoma samples (A).Supervised hierarchical clustering based upon the 133 differentially expressed genes between pancreatic and duodenal adenocarcinomas (B). Clinical characteristics are listed below the figure: age >65 y/o (black), male gender (black), poor differentiation (black), mucinous histology (black), T4 (black), N1 (black), and the presence of a precursor lesion such as an adenoma, dysplasia or pancreatic intraepithelial neoplasia (black). Overall survival by (C) tumor type and (D) gene expression grouping (group 1, n = 9; group 2, n = 13; group 3, n = 10).

We performed unsupervised hierarchical clustering upon all samples using all mRNA expression data (31,416 probesets), Figure 1a. This analysis identified two statistically different groups of periampullary carcinomas (p<0.01) with the ampullary carcinomas segregating into both groups. In order to further investigate these two ampullary subgroups and to directly compare ampullary carcinomas to non-ampullary periampullary carcinomas, we performed supervised hierarchical clustering analysis of the expression pattern of the genes (n = 133) that showed significant differences in expression between pancreatic and duodenal cases. This analysis identified three distinct groups (Figure 1b). Group 1 included all of the pancreatic carcinomas, and one duodenal carcinoma. This duodenal sample did not appear to be a misclassification as it demonstrated less than 1 mm invasion into the pancreas by histology and 100% tumor tissue on H&E review of the frozen tissue sample. Groups 2 and 3 included the remaining duodenal tumors, and all of the ampullary tumors. The two extrahepatic cholangiocarcinomas included in the study also clustered together in Group 2.

Review of the clinical histories found that the patients with ampullary and duodenal adenocarcinomas survived longer than patients with pancreatic or biliary adenocarcinomas (Figure 1c). However, the difference did not reach statistical significance (Logrank test for trend, p = 0.13). In contrast, we observed significant difference in overall survival when these same samples were analyzed according to their gene expression groupings (Figure 1d, Logrank test for trend, p = 0.02). Consistent with the preponderance of pancreatic tumors, the patients in group 1 of Figure 1A had poor outcomes. Interestingly, the patients whose tumors were categorized as being in group 2 by mRNA expression had similarly poor outcomes to those patients in group 1, whereas the patients in group 3 had dramatically improved overall survival in comparison to group 2. Both 5 year RFS (77% vs. 0%, p = 0.007) and 5 year OS (100% vs. 35%, p = 0.005) were significantly improved in group 3 as compared to group 2. When only the ampullary carcinomas were analyzed a similar trend of improved OS in group 3 ampullary carcinomas as compared to group 2 ampullary carcinomas was seen (p = 0.07, data not shown).

### Identification of Ampullary Subgroups

**Figure 2:**
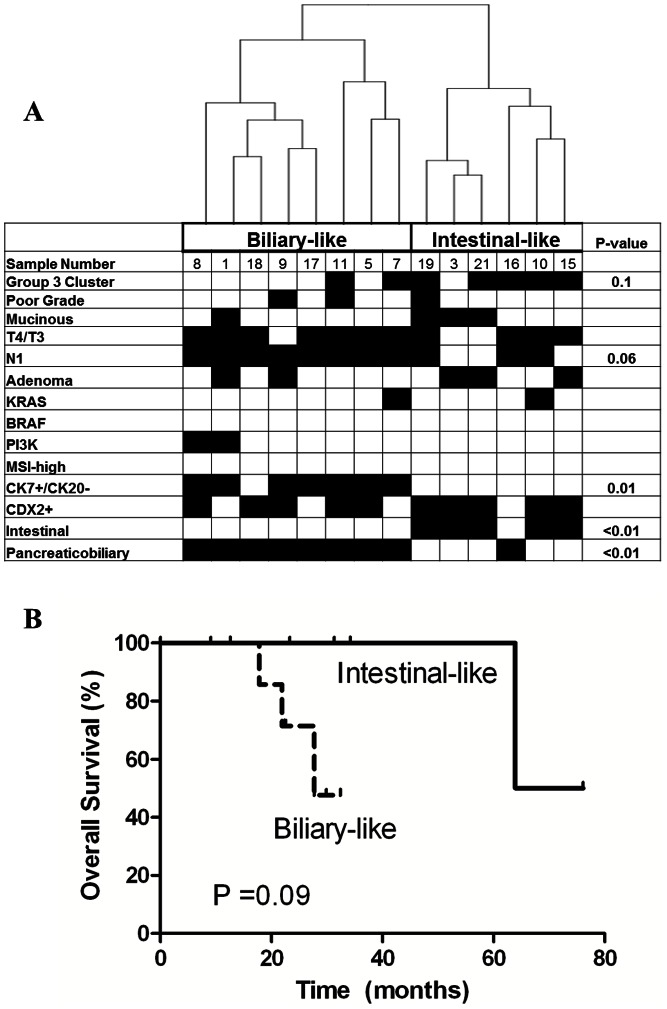
Unsupervised hierarchical clustering of ampullary adenocarcinoma samples, n = 14 (A).Clinical and molecular characteristics are listed below the figure: group 3 gene expression grouping (black), poor differentiation (black), mucinous histology (black), T4/T3 (black), N1 (black), presence of an adenoma (black), activation mutations in KRAS, BRAF, PI3K (black), MSI-high status (black), CK7+/CK20− (black), CDX-2+ (black), and histological subtype. Overall survival by gene expression derived biliary-like and intestinal-like ampullary subgroups (B).

To further validate our identification of two ampullary groups we performed unsupervised hierarchical clustering using all mRNA expression data (32,861 probesets) for the ampullary adenocarcinomas (n = 14), Figure 2a. This analysis identified 2 groups of ampullary carcinomas (p<0.01), which were similar to those seen from our prior analysis with one group (n = 6) containing 5 ampullary cases from group 3 and the other group (n = 8) containing 6 cases from group 2. Based upon the comparative gene expression grouping and the histology of these two groups we classified these groups as a biliary-like group and an intestinal-like group. The median OS in the intestinal-like group (70 months) was better than biliary-like group (28 months), though this difference did not reach statistical significance in this small cohort of patients (Figure 2b, p = 0.09).

A total of 234 genes showed significant differences in expression between these two ampullary groups (Table S1). In the intestinal-like ampullary group, a number of intestinal associated markers such as meprin A alpha [20], guanylate cyclase 2C [21], glycoprotein A33 [22], and CDX-1 [23] were found to be significantly upregulated compared to the biliary-like ampullary group. In contrast MUC1 [24], [25], a pancreaticobiliary mucin, was found upregulated in the biliary-like group. In addition, the second most upregulated gene in the biliary-like group was the anti-apoptotic gene clusterin (10 fold increase), which has been correlated with chemoresistance in pancreatic cancer. [26], [27] While NOTCH 2, a member of the Notch signaling pathway, was upregulated in the biliary-like ampullary group, Axin-2, a member of the WNT signaling pathway was upregulated in the intestinal-like ampullary group. Hepatocyte Nuclear Factor (HNF) 4α, which had previously been identified as a good prognostic marker in ampullary adenocarciomas, was found to be 2.2 fold upregulated in the intestinal-like subtype as compared to the biliary-like subtype, though with a FDR of 0.01 this was not significant [28].

In order to better understand the difference between these two groups, we examined a number of clinical, pathological, and previously-defined molecular characteristics (Figure 2a). Lymph node positivity was more common in the biliary-like group compared to the intestinal-like group (100% vs. 50%, p = 0.06). In contrast, there was no difference in other clinicopathological parameters such as the presence of a pre-existing adenoma or poorly differentiated histology. Molecular testing also showed no significant difference in the prevalence of mutations in *KRAS, BRAF,* or *PIK3CA* genes, or in MSI phenotype, between the two groups. Immunohistochemical analysis demonstrated positive CDX-2 expression in 83% of the intestinal-like group, but CDX-2 expression was also seen in 63% of the biliary-like group (p = 0.58). The factors best able to discriminate between the two ampullary subgroups were CK7+/CK20− immunohistochemical pattern (p<0.01) and histological subtype (p<0.01).

### Proteomic Profiling of Ampullary Adenocarcinomas

**Figure 3:**
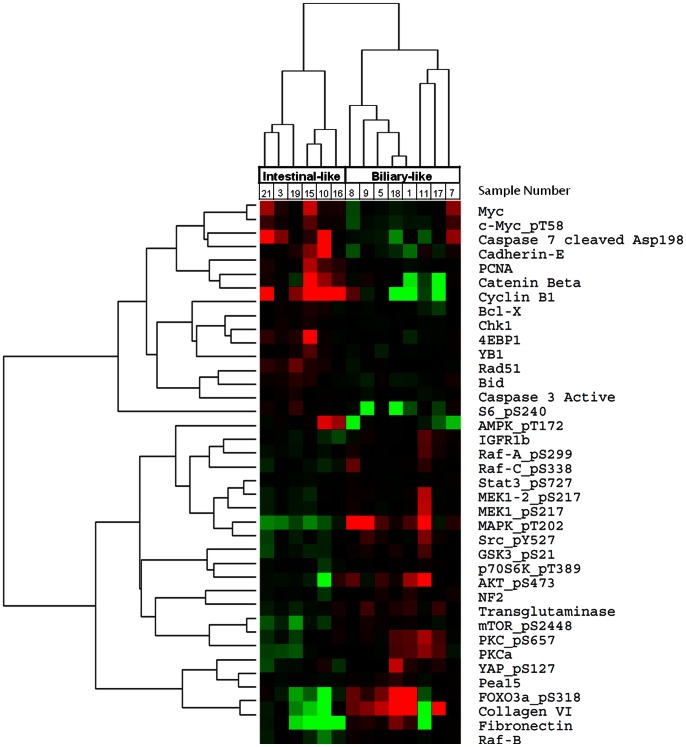
Unsupervised hierarchical clustering of the differentially expressed proteins (P<0.05) between gene expression derived biliary-like and intestinal-like ampullary subgroups.

To further characterize these two ampullary subgroups, quantitative analysis of the expression of 140 protein epitopes was performed on the 14 ampullary samples using the reverse phase protein array (RPPA) platform. Unsupervised hierarchical clustering of all analyzed proteins identified two nearly identical groups to our previously identified ampullary gene expression groups, with the exception of one sample (Figure S1). Supervised hierarchical clustering based upon the 38 differentially expressed proteins (p<0.05) identified the identical two groups as identified from our ampullary gene expression clustering, Figure 3. The biliary-like ampullary group, which showed a strong trend for shorter survival, showed increased expression of activation-specific markers in several kinase signaling pathways, including the PI3K-AKT (P-AKT Ser473, P-GSK3 Ser21, P-P70S6K T389, P-mTOR S2448, and P-FOXO3a), RAS-RAF-MEK-ERK (P-MAPK, P-MEK), and JAK-STAT (P-STAT3 S727) (Table S2). The intestinal-like ampullary group was characterized by increased expression of beta-catenin and E-Cadherin, suggestive of activation of the WNT pathway, and increased expression of both total and phospho-c-MYC. Correlation between protein expression and gene expression was seen for a number of proteins in the intestinal-like ampullary group with Spearman’s rank correlation >0.65 and p-value <0.01 for MYC, BID, YBX1, and CCNB1. For proteins within the biliary-like ampullary group, protein and gene expression levels correlated for members of the PI3K-AKT pathway: RPS6KB1 (Spearman’s rank correlation 0.67 and p-value 0.01) and PIK3R1 (Spearman’s rank correlation 0.56 and p-value 0.04).

### Validation of Ampullary Subtypes in an Independent Dataset

Since our mRNA profiling data and proteomic analysis of ampullary adenocarcinomas identified two prognostically distinct subgroups of ampullary adenocarcinomas we attempted to validate these findings. Due to the rarity of ampullary adenocarcinoma only one gene expression dataset of ampullary adenocarcinomas has been published. [28] In this dataset of 12 cases only 11 cases had outcome data. The application of our 234 gene classifier was able to identify two groups of ampullary adenocarcinomas (a poor prognosis biliary-like group of 2 cases and a good prognosis intestinal-like group of 10 cases) that demonstrated differing overall survival, P = 0.018 (Figure S2).

In addition, as our two ampullary subgroups could be histologically categorized as either an intestinal subtype or a pancreaticobiliary subtype, we examined the association of histological subtypes with prognosis in an independent cohort of 80 resected ampullary adenocarcinomas. The clinicopahological features of intestinal, pancreaticobiliary and mixed histologic subtypes of ampullary adenocarcinomas are listed in Table 1. Cases with an intestinal histological subtype were more likely to be node negative (p<0.01), have a lower T stage (p<0.01), an associated ampullary adenoma (p<0.01), a non-CK7+/CK20− cytokeratin profile, (p = 0.03), and have not received adjuvant chemotherapy (p = 0.04) compared to the pancreaticobiliary subtype. CDX-2 expression was more common in the intestinal subtype as compared to a pancreaticobiliary subtype, though this did not reach statistical significance, p = 0.07.

**Table 1:** Patient and Tumor Characteristics of Ampullary Validation Dataset.

	Intestinal		Pancreaticobiliary		Mixed	
	Pt. No.	%	Pt. No.	%	Pt. No.	%
Histological subtype	24	30	32	40	24	30
Median age, years (range)	66 (30–87)		63 (28–83)		64 (37–77)	
Female gender	12	50	11	34	11	46
T Stage						
T1	10	42	2	6	4	17
T2	14	58	9	28	8	33
T3	0	0	19	59	11	46
T4	0	0	2	6	0	0
N Stage						
N0	19	79	8	25	11	46
N1	5	21	24	75	13	54
Differentiation						
Well	5	21	3	9	0	0
Moderate	13	54	14	44	16	67
Poor	6	25	15	47	8	33
Margin Status						
R0	24	100	28	88	23	96
R1	0	0	4	13	1	4
Mucinous	0	0	2	6	1	4
Ampullary adenoma	17	71	2	6	15	63
Adjuvant Treatment						
Systemic Chemotherapy	5	21	19	59	12	50
Radiation Therapy	5	21	17	53	10	42
CDX-2+	14	58	9	28	9	38
CK7+/CK20−	8	33	22	69	11	46

**Figure 4:**
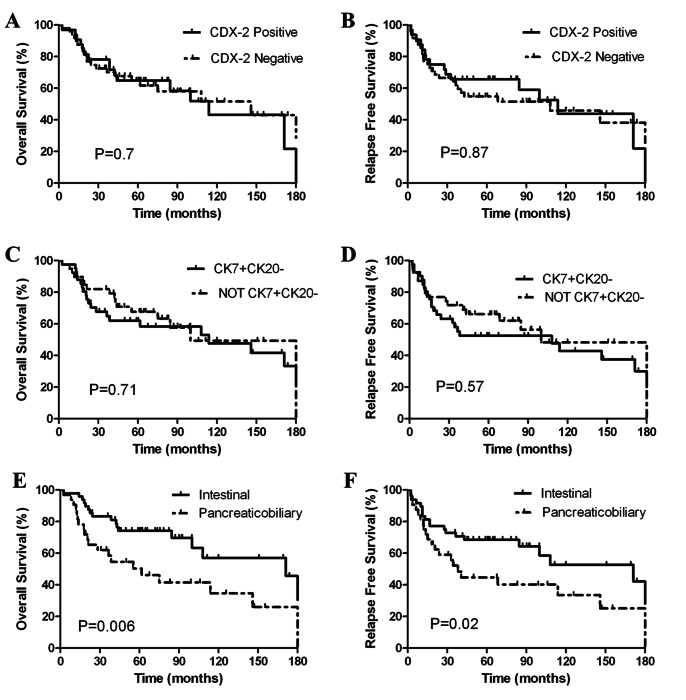
Overall survival and relapse-free survival for the 80 patient ampullary dataset stratified by (A,B) CDX-2 expression status, (C,D) CK7+/CK20− expression status, and (E,F) histological subtype, respectively.

Neither the cytokeratin 7+/20− expression pattern, nor the expression of CDX-2 was correlated with an improved RFS or OS (Figure 4a–4d). In contrast, histological subtype significantly correlated with survival with intestinal, mixed, and pancreaticobiliary subtypes demonstrating a 5-year OS of 70%, 77%, and 50%, and a 5-year RFS of 71%, 66%, and 45%, respectively. As intestinal and mixed histological subtypes had similar outcomes, these two groups when combined demonstrated significant improvements in OS (171 months vs. 62 months, p = 0.006) and RFS (171 months vs. 38 months, p = 0.02) when compared to the pancreaticobiliary subtype (Figure 4e–4f). Although no factors were significantly associated with RFS in multivariate analysis (data not shown), the pancreaticobiliary histological subtype was significantly associated with worse OS by multivariate analysis, Table 2.

**Table 2:** Univariate and Multivariate Analysis of Factors Associated with Overall Survival.

	Univariate			Multivariate		
	HR	95% CI	P-value	HR	95% CI	P-value
Age	1.00	0.98–1.03	0.84			
Poorly Differentiated	0.72	0.27–1.9	0.51			
T3/T4	2.74	0.99–7.6	0.05	1.94	0.66–5.71	0.23
Lymph Node Positive	1.28	0.67–2.44	0.46			
Positive Margins	1.63	0.38–6.93	0.51			
Adjuvant Treatment	1.02	0.52–1.99	0.95			
Pancreaticobiliary Subtype	2.47	1.28–4.79	0.007	2.09	1.03–4.27	0.04
CDX-2 Positive	1.14	0.59–2.21	0.7			
CK7+/CK20−	1.14	0.58–2.22	0.71			

## Discussion

In this study, we used comparative molecular and histological analyses of periampullary adenocarcinomas to gain insights into ampullary adenocarcinoma. Our gene expression analysis has demonstrated a molecular distinction between ampullary and pancreatic adenocarcinomas. More importantly, our gene expression and proteomic analysis identified two prognostically relevant subgroups of ampullary adenocarcinomas that can be histologically categorized as either intestinal-like or pancreaticobiliary-like ampullary subtypes. Univariate and multivariate analysis of an independent validation cohort demonstrated that histologic subtype is an independent prognostic factor in patients with ampullary adenocarcinoma. Patients with intestinal subtype of ampullary adenocarcinoma have better OS than those with pancreaticobiliary subtype, which is characterized by the activation of several targetable pathways. Thus our findings support both the biological and clinical significance of classifying ampullary adenocarcinomas into two distinct subtypes, and suggest potential subtype-specific therapeutic strategies.

In this study, the samples selected for gene expression and proteomic analysis were required to meet strict inclusion criteria to minimize any impact of cancer misclassification and dilution of results through inclusion of non-carcinoma tissue. By classifying the ampullary carcinomas samples as unknowns and comparing them to pathologically verified duodenal, biliary and pancreatic carcinoma samples, we have attempted to molecularly characterize the epithelium of origin of the histologically diverse ampullary adenocarcinomas. Our gene expression array data clearly show that ampullary adenocarcinomas have a different gene expression profile compared to pancreatic adenocarcinomas. Thus, our data suggests that ampullary adenocarcinomas do not arise from the ampullo-pancreatic ductal epithelial cells in the ampulla of Vater. Though our study was limited by the presence of only two extrahepatic biliary cases, these cases grouped with our ampullary samples that displayed a pancreaticobiliary morphology. As our ampullary and pancreatic adenocarcinomas were clearly distinct, we feel this group is best classified as a biliary-like subgroup of ampullary adenocarcinoma.

As post-translational modifications can lead to marked discordance between mRNA levels and protein function [29], [30], we directly examined the expression of proteins and phospho-proteins in a number of known oncogenic signaling pathways. Our RPPA analysis was able to recapitulate the molecular heterogeneity identified by whole-genomic transcriptional profiling and concordantly identified two prognostically relevant subgroups of ampullary adenocarcinomas. RPPA analysis demonstrated marked activation of both the PI3K-AKT and RAS-RAF-MAPK pathways in our poor prognosis pancreaticobiliary-like ampullary subgroup. Of note, both ampullary adenocarcinoma samples with mutations in *PIK3CA* in this cohort of patients occurred in the pancreaticobiliary-like subgroup. These findings support the rationale for functional testing of inhibitors against these pathways in biliary-like ampullary carcinomas. In the intestinal-like subgroup a number of intestinal-specific genes were upregulated, which suggests that this subgroup of ampullary carcinomas may arise from the overlying ampullo-duodenal epithelium of the ampulla of Vater.

Since prior studies have inconsistently reported the prognostic relevance of CDX-2 expression, CK7+/CK20− expression, and histological subtypes, we examined these markers. [4], [5], [9]–[13] We found that CDX-2 expression status represented an imperfect marker in comparison to our gene expression groupings. CK7+/CK20− expression pattern and histological subtype were the two factors most closely correlated with our ampullary gene expression groupings. In our validation cohort, only histological subtype demonstrated a consistent prognostic impact and was an independent prognostic factor for OS in patients with ampullary adenocarcinoma. These results are consistent with the recently reported ESPAC-3 periampullary randomized study in which the intestinal as opposed to the pancreaticobiliary subtype of ampullary adenocarcinomas demonstrated an improved DFS (45.7 vs. 20.6 m, p = 0.01) but not OS (56 vs. 43.1 m, p = 0.28). [8] At present reporting the histological subtype of ampullary adenocarcinomas is not standard, as was demonstrated by the ESPAC-3 study in which only 45% of all ampullary adenocarcinomas had histological subtype reported. Our data does differ from a prior report that found statistical significance with the use of CDX-2 and CDX-1 staining in 53 resected ampullary carcinoma patients. [13] However, our data is consistent with two subsequent cohorts of 53 and 71 patients that did not identify CDX-2 expression as a prognostic marker [5], [12].

Recently, our clinical approach for both metastatic duodenal and biliary adenocarcinomas has improved with the publication of a phase II study evaluating capecitabine and oxaliplatin in small bowel adenocarcinoma and a phase III study evaluating gemcitabine and cisplatin in biliary adenocarcinoma. [31], [32] Interestingly, as a testament to the uncertainty of how to approach ampullary adenocarcinomas, both studies included adenocarcinomas of the ampulla of Vater. The recently completed ESPAC-3 study evaluated the role of adjuvant therapy for ampullary adenocarcinomas and found no difference with regard to the use of either gemcitabine or 5-FU in the adjuvant setting. [8] Though our findings do not provide a direct link between the expression profiling or histological subtype of ampullary adenocarcinoma and chemotherapy benefit, we do feel that histological subtype deserves further study as a potential marker to better select patients for adjuvant therapy and possibly as a means to optimally select chemotherapy, 5-FU-based as opposed to gemcitabine-based.

There are a number of limitations to this study. Our gene expression analysis was limited by the availability of a small number of frozen tumor samples and should be viewed as an exploratory analysis that requires validation in other larger datasets. Recently a multicenter rare cancer genome project to investigate periampullary adenocarcinomas has been initiated and data from this effort will represent a potential validation cohort in the future. The consistent correlations between gene expression, protein expression, histology, and outcome do support our findings. In addition the ability to validate our findings in a large separate cohort of ampullary adenocarcinomas lends support to our gene expression analysis. The classification of the origin of periampullary adenocarcinomas is primarily based upon the clinical and pathological determination of the epicenter of the tumor. This classification can be challenging in large, locally advanced tumors. To minimize misclassification, we relied upon full resection specimen review for each sample and strict criteria in regards to tumor percentage and RNA quality. However, the potential to more precisely classify these tumors lends further support to future efforts, as outlined in this study, to classify the tissue of origin of periampullary cancers based on gene expression profiling. Such an approach may actually better reflect the true tissue of origin and expected natural history for each cancer case.

The limited availability of frozen tissue samples and the rarity of non-pancreatic periampullary adenocarcinomas have limited both the molecular and clinical understanding of these cancers. This study improves our understanding of ampullary adenocarcinomas and distinguishes these cancers from pancreatic adenocarcinomas. Ampullary adenocarcinomas demonstrate both molecular and clinical heterogeneity. Further research into the treatment implications of these findings is needed.

## Supporting Information

Figure S1 **Unsupervised hierarchical clustering of all proteins from the 14 ampullary adenocarcinoma samples.**
(TIF)

Figure S2 Clustering of 12 additional ampullary adenocarcinoma samples (Ehehalt et al.) using the 234 differentially expressed genes identifies a two sample biliary-like subgroup and a 10 patient intestinal-like subgroup (A). Overall survival for the 11 cases with available outcome data (B).
(TIF)

Table S1 **The 234 differentially expressed genes between intestinal-like and biliary-like ampullary subtypes.**
(DOCX)

Table S2 **Quantitative protein expression data between intestinal-like and biliary-like ampullary subtypes.**
(DOCX)

Methods S1 **Methodology for tissue microarray construction, immunohistochemical analysis, microsatellite instability determination, and DNA mutation analysis.**
(DOCX)
