# Correction: Gene Expression Profiling of Ampullary Carcinomas Classifies Ampullary Carcinomas into Biliary-Like and Intestinal-Like Subtypes That Are Prognostic of Outcome

**DOI:** 10.1371/annotation/6afdd046-e1a7-4421-b7ec-0c9492ce2544

**Published:** 2013-07-03

**Authors:** Michael J. Overman, Jiexin Zhang, Scott Kopetz, Michael Davies, Zhi-Qin Jiang, Katherine Stemke-Hale, Petra Rümmele, Christian Pilarsky, Robert Grützmann, Stanley Hamilton, Rosa Hwang, James L. Abbruzzese, Gauri Varadhachary, Bradley Broom, Huamin Wang

The name of the fifth author was given incorrectly. The correct name is: Zhi-Qin Jiang. The correct citation is: Overman MJ, Zhang J, Kopetz S, Davies M, Jiang Z-Q, et al. (2013) Gene Expression Profiling of Ampullary Carcinomas Classifies Ampullary Carcinomas into Biliary-Like and Intestinal-Like Subtypes That Are Prognostic of Outcome. PLoS ONE 8(6): e65144. doi:10.1371/journal.pone.0065144. The correct abbreviation in Citations is: ZQJ. 

