# Supplementary figures and images for: Gene Expression Profiling of Ampullary Carcinomas Classifies Ampullary Carcinomas into Biliary-Like and Intestinal-Like Subtypes That Are Prognostic of Outcome
Source: PLoS One. 2013 Jun 11;8(6):e65144. doi: 10.1371/journal.pone.0065144 (PMC3679143; doi:10.1371/journal.pone.0065144)

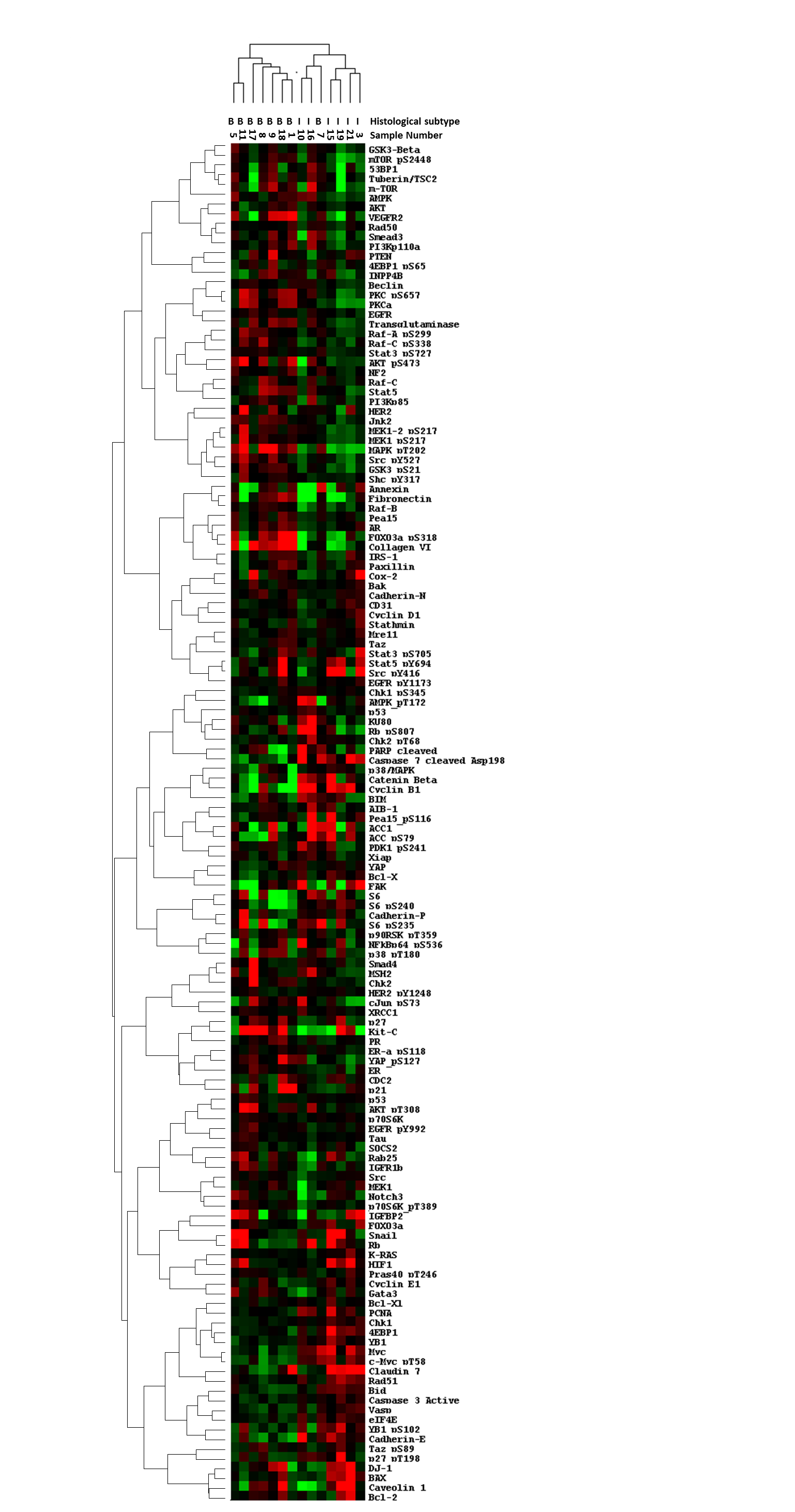

Supplement: Figure S1 — Unsupervised hierarchical clustering of all proteins from the 14 ampullary adenocarcinoma samples. (TIF) [file pone.0065144.s001.tif]

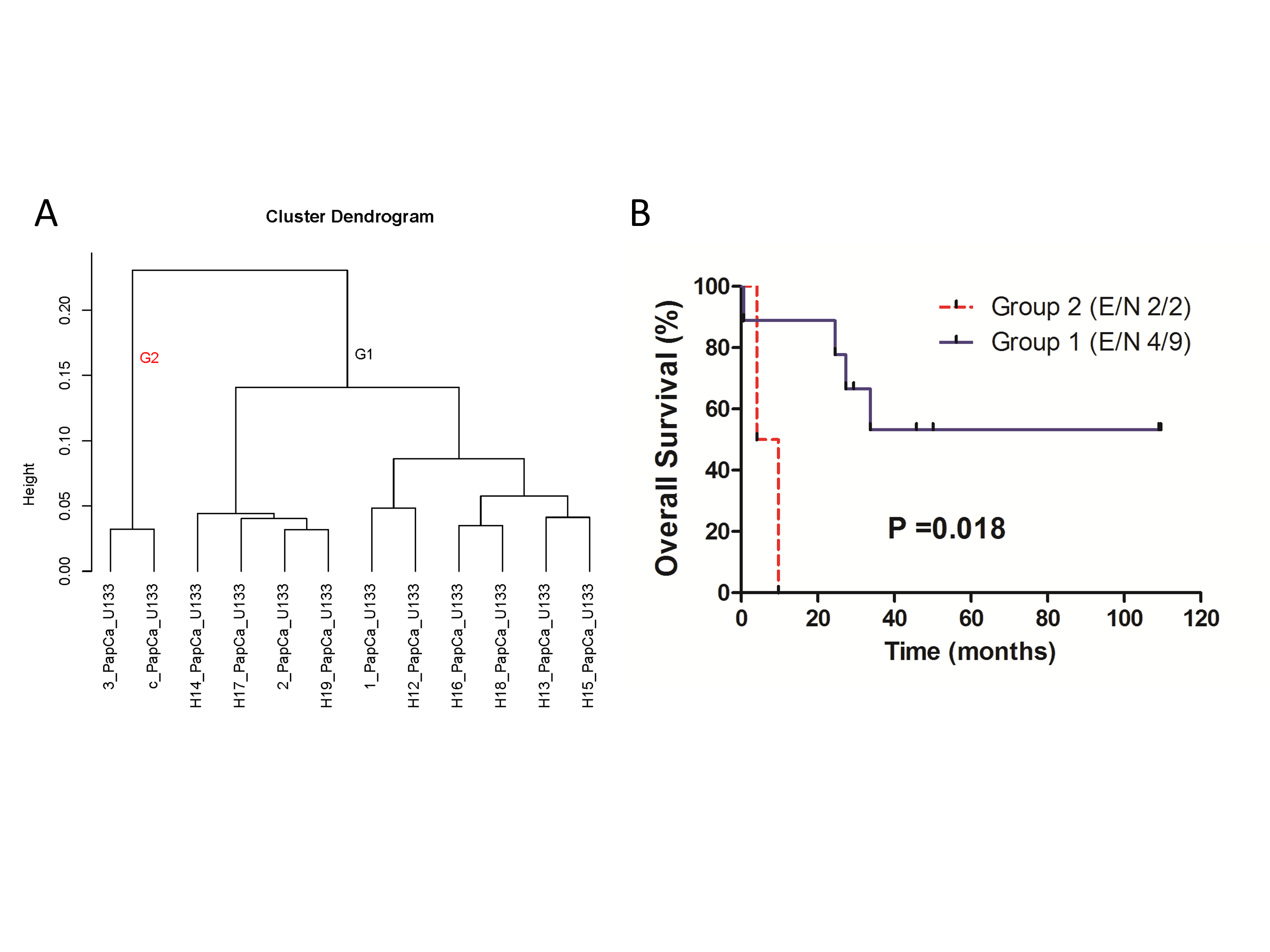

Supplement: Figure S2 — Clustering of 12 additional ampullary adenocarcinoma samples (Ehehalt et al.) using the 234 differentially expressed genes identifies a two sample biliary-like subgroup and a 10 patient intestinal-like subgroup (A). Overall survival for the 11 cases with available outcome data (B). (TIF) [file pone.0065144.s002.tif]
