# Supplementary material for: Gene Expression Profiling of Ampullary Carcinomas Classifies Ampullary Carcinomas into Biliary-Like and Intestinal-Like Subtypes That Are Prognostic of Outcome
Source: PLoS One. 2013 Jun 11;8(6):e65144. doi: 10.1371/journal.pone.0065144 (PMC3679143; doi:10.1371/journal.pone.0065144)
Supplement: Table S1 — The 234 differentially expressed genes between intestinal-like and biliary-like ampullary subtypes. (DOCX) [file pone.0065144.s003.docx]

Table S1: The 234 differentially expressed genes between intestinal-like and biliary-like ampullary subtypes

| Gene Symbol | Gene Name | Fold Change | P-value | Probe-set ID |
| --- | --- | --- | --- | --- |
| UBE2N | ubiquitin-conjugating enzyme E2N (UBC13 homolog, yeast) | 1.62 | 7.34E-08 | 201523_x_at |
| YPEL2 | yippee-like 2 (Drosophila) | 0.37 | 8.10E-08 | 227020_at |
| CDC2 | Cell division cycle 2, G1 to S and G2 to M | 1.69 | 1.43E-07 | 231534_at |
| CASC4 | cancer susceptibility candidate 4 | 0.62 | 1.54E-07 | 1552719_at |
| PANK1 | pantothenate kinase 1 | 4.51 | 2.55E-07 | 226649_at |
| KRT18 | keratin 18 | 2.13 | 2.79E-07 | 201596_x_at |
| SLC27A1 | solute carrier family 27 (fatty acid transporter), member 1 | 0.47 | 3.68E-07 | 226728_at |
| DPEP1 | dipeptidase 1 (renal) | 2.77 | 6.56E-07 | 205983_at |
| KIAA1641 | KIAA1641 | 0.36 | 9.24E-07 | 220940_at |
| CDX1 | caudal type homeo box transcription factor 1 | 2.46 | 1.11E-06 | 206430_at |
| UGCG | UDP-glucose ceramide glucosyltransferase | 0.46 | 1.72E-06 | 204881_s_at |
| --- | Homo sapiens, clone IMAGE:5188742, mRNA | 1.94 | 1.89E-06 | 1559538_at |
| STX3A | syntaxin 3A | 1.56 | 2.20E-06 | 216985_s_at |
| PLA2G12B | phospholipase A2, group XIIB | 3.68 | 2.25E-06 | 231009_at |
| C10orf125 | chromosome 10 open reading frame 125 | 2.01 | 2.38E-06 | 230259_at |
| CALM3 | calmodulin 3 (phosphorylase kinase, delta) | 1.49 | 2.73E-06 | 200622_x_at |
| LOC157860 | hypothetical protein LOC157860 | 2.30 | 2.88E-06 | 232040_at |
| EFNA5 | Nuclear RNA-binding protein, putative | 0.12 | 3.10E-06 | 1559360_at |
| STOX2 | storkhead box 2 | 0.13 | 3.24E-06 | 231969_at |
| NSE1 | Family with sequence similarity 84, member A | 3.65 | 3.52E-06 | 231439_at |
| RBM19 | RNA binding motif protein 19 | 1.22 | 3.86E-06 | 205115_s_at |
| VDR | vitamin D (1,25- dihydroxyvitamin D3) receptor | 3.03 | 4.35E-06 | 204255_s_at |
| FLJ25067 | hypothetical protein FLJ25067 | 1.41 | 4.60E-06 | 243507_s_at |
| YY1 | YY1 transcription factor | 1.25 | 4.61E-06 | 201902_s_at |
| CCND2 | cyclin D2 | 3.65 | 4.73E-06 | 200951_s_at |
| YPEL2 | yippee-like 2 (Drosophila) | 0.43 | 4.76E-06 | 1556420_s_at |
| --- | --- | 0.67 | 4.82E-06 | 241336_at |
| WSB1 | WD repeat and SOCS box-containing 1 | 0.61 | 4.83E-06 | 227501_at |
| POGZ | pogo transposable element with ZNF domain | 0.57 | 7.33E-06 | 212153_at |
| HRPT2 | Cell division cycle 73, Paf1/RNA polymerase II complex component, homolog (S. cerevisiae) | 0.58 | 7.36E-06 | 222320_at |
| PTBP1 | polypyrimidine tract binding protein 1 | 1.58 | 7.42E-06 | 212016_s_at |
| LYK5 | protein kinase LYK5 | 0.79 | 7.58E-06 | 52169_at |
| MUC1 | mucin 1, transmembrane | 0.22 | 8.32E-06 | 213693_s_at |
| --- | --- | 2.13 | 8.54E-06 | 234954_at |
| DSG4 | desmoglein 4 | 1.23 | 8.65E-06 | 1561330_at |
| CDC6 | CDC6 cell division cycle 6 homolog (S. cerevisiae) | 2.62 | 8.71E-06 | 203968_s_at |
| PTBP2 | Polypyrimidine tract binding protein 2 | 0.44 | 9.13E-06 | 1560271_at |
| AXIN2 | axin 2 (conductin, axil) | 3.38 | 9.63E-06 | 222696_at |
| PTK9 | PTK9 protein tyrosine kinase 9 | 1.97 | 1.00E-05 | 214007_s_at |
| UGCG | UDP-glucose ceramide glucosyltransferase | 0.39 | 1.03E-05 | 221765_at |
| MKI67 | antigen identified by monoclonal antibody Ki-67 | 2.01 | 1.03E-05 | 212020_s_at |
| SUHW4 | Suppressor of hairy wing homolog 4 (Drosophila) | 0.44 | 1.23E-05 | 236153_at |
| PSME3 | proteasome (prosome, macropain) activator subunit 3 (PA28 gamma; Ki) | 1.31 | 1.24E-05 | 209852_x_at |
| FLJ20719 /// LOC200030 /// MGC8902 /// AE01 /// AG1 /// LOC440675 | hypothetical protein FLJ20719 /// hypothetical protein LOC200030 /// hypothetical protein MGC8902 /// AE01 mRNA /// AG1 protein /// hypothetical LOC440675 | 0.60 | 1.25E-05 | 201103_x_at |
| USP30 | ubiquitin specific peptidase 30 | 1.93 | 1.35E-05 | 223602_at |
| CDC2 | cell division cycle 2, G1 to S and G2 to M | 2.75 | 1.35E-05 | 210559_s_at |
| FLJ20719 /// MGC8902 /// AE01 /// AG1 /// LOC440675 | hypothetical protein FLJ20719 /// hypothetical protein MGC8902 /// AE01 mRNA /// AG1 protein /// hypothetical LOC440675 | 0.64 | 1.40E-05 | 213612_x_at |
| CDC2 | cell division cycle 2, G1 to S and G2 to M | 2.35 | 1.49E-05 | 203214_x_at |
| C14orf2 | chromosome 14 open reading frame 2 | 1.64 | 1.68E-05 | 210532_s_at |
| FLJ20641 | hypothetical protein FLJ20641 | 2.36 | 1.68E-05 | 227928_at |
| CCDC5 | coiled-coil domain containing 5 (spindle associated) | 2.25 | 1.69979E-05 | 225297_at |
| TTBK2 | tau tubulin kinase 2 | 0.42 | 1.74038E-05 | 213922_at |
| --- | Hypothetical LOC388388 | 1.70 | 1.81431E-05 | 214349_at |
| PCGF5 | polycomb group ring finger 5 | 1.46 | 1.8174E-05 | 227935_s_at |
| --- | CDNA: FLJ22256 fis, clone HRC02860 | 0.24 | 1.85606E-05 | 227955_s_at |
| NUP160 | nucleoporin 160kDa | 1.37 | 1.86378E-05 | 214962_s_at |
| ARGBP2 | Sorbin and SH3 domain containing 2 | 0.20 | 1.87429E-05 | 238751_at |
| FLJ43663 | Hypothetical protein FLJ43663 | 0.50 | 1.89073E-05 | 239901_at |
| SNX24 | sorting nexing 24 | 0.47 | 1.9052E-05 | 222716_s_at |
| VIL1 | villin 1 | 5.05 | 1.92284E-05 | 205506_at |
| C19orf32 | chromosome 19 open reading frame 32 | 3.69 | 2.00682E-05 | 226597_at |
| SYNPO2 | synaptopodin 2 | 0.05 | 2.01471E-05 | 227662_at |
| ECHS1 | enoyl Coenzyme A hydratase, short chain, 1, mitochondrial | 2.16 | 2.02596E-05 | 201135_at |
| CHEK1 | CHK1 checkpoint homolog (S. pombe) | 3.52 | 2.05978E-05 | 205393_s_at |
| TPMT | thiopurine S-methyltransferase | 1.74 | 2.18716E-05 | 203672_x_at |
| MEIS2 | Meis1, myeloid ecotropic viral integration site 1 homolog 2 (mouse) | 0.13 | 2.1885E-05 | 207480_s_at |
| FAM84A /// LOC400944 | family with sequence similarity 84, member A /// hypothetical LOC400944 | 8.04 | 2.23294E-05 | 225667_s_at |
| HMMR | hyaluronan-mediated motility receptor (RHAMM) | 2.51 | 2.24797E-05 | 209709_s_at |
| NXF1 | nuclear RNA export factor 1 | 0.76 | 2.26985E-05 | 208922_s_at |
| RBPMS | RNA binding protein with multiple splicing | 0.25 | 2.29046E-05 | 241897_at |
| C10orf45 | chromosome 10 open reading frame 45 | 0.27 | 2.35376E-05 | 223059_s_at |
| RAP2B | RAP2B, member of RAS oncogene family | 0.57 | 2.37525E-05 | 213923_at |
| --- | CDNA clone IMAGE:4821815 | 0.23 | 2.44523E-05 | 243974_at |
| OSBPL9 | Oxysterol binding protein-like 9 | 0.50 | 2.5456E-05 | 236472_at |
| --- | --- | 1.51 | 2.59749E-05 | 217209_at |
| KRT8 | keratin 8 /// keratin 8 | 2.23 | 2.69835E-05 | 209008_x_at |
| ATXN7L1 /// ATXN7L4 | ataxin 7-like 1 /// ataxin 7-like 4 | 1.28 | 2.70263E-05 | 214343_s_at |
| --- | Transcribed locus | 1.35 | 2.7722E-05 | 235993_at |
| BRCA2 | breast cancer 2, early onset | 1.47 | 2.86559E-05 | 214727_at |
| PTGIS | prostaglandin I2 (prostacyclin) synthase /// prostaglandin I2 (prostacyclin) synthase | 0.17 | 2.94779E-05 | 208131_s_at |
| --- | --- | 0.21 | 2.95355E-05 | 1554007_at |
| ARID4B | AT rich interactive domain 4B (RBP1- like) | 0.60 | 3.05182E-05 | 235959_at |
| TLE3 | transducin-like enhancer of split 3 (E(sp1) homolog, Drosophila) | 1.23 | 3.10721E-05 | 206472_s_at |
| BBX | Bobby sox homolog (Drosophila) | 0.39 | 3.118E-05 | 1557240_a_at |
| PTBP2 | polypyrimidine tract binding protein 2 | 0.46 | 3.1497E-05 | 218683_at |
| RFC5 | replication factor C (activator 1) 5, 36.5kDa | 1.50 | 3.17371E-05 | 203210_s_at |
| PLK4 | polo-like kinase 4 (Drosophila) | 1.75 | 3.19606E-05 | 204887_s_at |
| KIAA0582 | KIAA0582 | 0.67 | 3.20979E-05 | 239442_at |
| LOC200030 | hypothetical protein LOC200030 | 0.68 | 3.22162E-05 | 229447_x_at |
| FLJ20641 | hypothetical protein FLJ20641 | 1.87 | 3.23299E-05 | 220060_s_at |
| TERF2 | telomeric repeat binding factor 2 | 1.33 | 3.25532E-05 | 229790_at |
| C18orf24 | chromosome 18 open reading frame 24 | 1.28 | 3.29608E-05 | 217640_x_at |
| MYOZ3 | myozenin 3 | 0.80 | 3.33209E-05 | 213955_at |
| GUCY2C | guanylate cyclase 2C (heat stable enterotoxin receptor) | 8.96 | 3.33597E-05 | 206312_at |
| CDA08 | T-cell immunomodulatory protein | 1.33 | 3.3452E-05 | 239044_at |
| PIP5K2B | Phosphatidylinositol-4-phosphate 5-kinase, type II, beta | 0.70 | 3.37267E-05 | 201080_at |
| TRAF3IP3 | TRAF3 interacting protein 3 | 1.38 | 3.37374E-05 | 215275_at |
| FLJ13710 | hypothetical protein FLJ13710 | 0.21 | 3.38798E-05 | 222835_at |
| LOC283768 /// LOC388080 /// LOC388189 /// LOC390535 /// LOC400304 /// LOC440234 /// DKFZp434P162 | hypothetical LOC283768 /// similar to hypothetical protein /// similar to hypothetical protein /// golgi autoantigen, golgin family member /// similar to hypothetical protein /// similar to hypothetical protein /// hypothetical protein DKFZp434P162 | 0.42 | 3.39112E-05 | 213737_x_at |
| --- | CDNA clone IMAGE:5759225 | 1.71 | 3.4238E-05 | 230485_at |
| AG1 | AG1 protein | 0.60 | 3.45455E-05 | 226740_x_at |
| PCDHB16 | protocadherin beta 16 | 0.23 | 3.54637E-05 | 232099_at |
| GABRA2 | gamma-aminobutyric acid (GABA) A receptor, alpha 2 | 1.30 | 3.61386E-05 | 1554308_s_at |
| C20orf17 | Chromosome 20 open reading frame 17 | 0.37 | 3.71265E-05 | 235616_at |
| DIABLO | diablo homolog (Drosophila) | 1.27 | 3.71883E-05 | 219350_s_at |
| GPR172A | G protein-coupled receptor 172A | 1.95 | 3.79288E-05 | 222155_s_at |
| SPBC25 | spindle pole body component 25 homolog (S. cerevisiae) | 2.64 | 3.98166E-05 | 209891_at |
| EIF2B2 | eukaryotic translation initiation factor 2B, subunit 2 beta, 39kDa | 1.70 | 3.98193E-05 | 202461_at |
| MTHFR | 5,10-methylenetetrahydrofolate reductase (NADPH) | 0.70 | 4.02136E-05 | 226929_at |
| --- | Full-length cDNA clone CS0DM011YC22 of Fetal liver of Homo sapiens (human) | 0.32 | 4.16327E-05 | 221973_at |
| FAM45B /// FAM45A | family with sequence similarity 45, member B /// family with sequence similarity 45, member A | 1.38 | 4.16494E-05 | 222955_s_at |
| MEP1A | meprin A, alpha (PABA peptide hydrolase) | 10.85 | 4.2427E-05 | 206000_at |
| TSC1 | tuberous sclerosis 1 | 0.66 | 4.34134E-05 | 209390_at |
| CLDN3 | claudin 3 | 5.79 | 4.41374E-05 | 203953_s_at |
| TDP1 | tyrosyl-DNA phosphodiesterase 1 | 1.56 | 4.50482E-05 | 219715_s_at |
| PARVA | parvin, alpha | 0.65 | 4.82953E-05 | 1563458_at |
| TXNL2 | thioredoxin-like 2 | 1.83 | 4.8615E-05 | 209080_x_at |
| POLA2 | polymerase (DNA directed), alpha 2 (70kD subunit) | 1.54 | 4.9335E-05 | 204441_s_at |
| ACSL5 | acyl-CoA synthetase long-chain family member 5 | 3.83 | 4.96206E-05 | 222592_s_at |
| DIAPH3 | diaphanous homolog 3 (Drosophila) | 1.68 | 4.98729E-05 | 232596_at |
| C10orf78 | chromosome 10 open reading frame 78 | 1.34 | 4.98845E-05 | 238794_at |
| MYEF2 | myelin expression factor 2 | 0.22 | 5.04758E-05 | 232676_x_at |
| PTTG3 | pituitary tumor-transforming 3 | 1.45 | 5.27888E-05 | 208511_at |
| PDGFC | Platelet derived growth factor C | 0.26 | 5.29405E-05 | 1563467_at |
| KIAA0934 | KIAA0934 | 0.40 | 5.33937E-05 | 1560763_at |
| AMSH-LP | associated molecule with the SH3 domain of STAM (AMSH) like protein | 2.44 | 5.39112E-05 | 227606_s_at |
| LOC390980 | similar to Zinc finger protein 264 | 0.63 | 5.43676E-05 | 238436_s_at |
| EIF2S1 | eukaryotic translation initiation factor 2, subunit 1 alpha, 35kDa | 2.00 | 5.50295E-05 | 201144_s_at |
| SCD | stearoyl-CoA desaturase (delta-9-desaturase) | 1.65 | 5.5321E-05 | 200831_s_at |
| MPHOSPH9 | M-phase phosphoprotein 9 | 1.24 | 5.54076E-05 | 237158_s_at |
| UGCG | UDP-glucose ceramide glucosyltransferase | 0.48 | 5.55055E-05 | 224967_at |
| CDKN3 | cyclin-dependent kinase inhibitor 3 (CDK2-associated dual specificity phosphatase) | 2.64 | 5.68836E-05 | 1555758_a_at |
| --- | --- | 1.17 | 5.69935E-05 | 217333_at |
| DAPK1 | Death-associated protein kinase 1 | 0.30 | 5.70433E-05 | 239162_at |
| LOC344595 | hypothetical LOC344595 | 0.71 | 5.74779E-05 | 239466_at |
| --- | --- | 8.31 | 5.87683E-05 | 242660_at |
| FLJ20315 | hypothetical protein FLJ20315 | 3.42 | 5.87897E-05 | 218704_at |
| KIAA1641 | KIAA1641 | 0.44 | 5.99443E-05 | 214723_x_at |
| MMP14 | matrix metallopeptidase 14 (membrane-inserted) | 1.24 | 6.04814E-05 | 217279_x_at |
| MLPH | melanophilin | 0.35 | 6.10165E-05 | 218211_s_at |
| KIAA0586 | KIAA0586 | 1.28 | 6.33987E-05 | 205631_at |
| C20orf17 | Chromosome 20 open reading frame 17 | 0.33 | 6.39955E-05 | 244521_at |
| ATAD2 | ATPase family, AAA domain containing 2 | 2.33 | 6.50571E-05 | 218782_s_at |
| DNAJC9 | DnaJ (Hsp40) homolog, subfamily C, member 9 | 2.35 | 6.54677E-05 | 213092_x_at |
| TXNL2 | thioredoxin-like 2 | 1.58 | 6.60689E-05 | 214205_x_at |
| --- | Transcribed locus | 0.64 | 6.61687E-05 | 236592_at |
| PRAP1 | proline-rich acidic protein 1 | 3.18 | 6.6642E-05 | 243669_s_at |
| PPAP2B | phosphatidic acid phosphatase type 2B | 0.45 | 6.76445E-05 | 212226_s_at |
| ZNF42 | zinc finger protein 42 (myeloid-specific retinoic acid-responsive) | 0.56 | 6.80449E-05 | 40569_at |
| FLJ10980 | Hypothetical protein FLJ10980 | 0.48 | 6.8863E-05 | 236237_at |
| KIAA0152 | KIAA0152 | 1.82 | 6.90241E-05 | 200616_s_at |
| SRPK1 | SFRS protein kinase 1 | 1.38 | 6.9221E-05 | 202199_s_at |
| ZNF134 | Zinc finger protein 134 (clone pHZ-15) | 0.60 | 6.98209E-05 | 227729_at |
| SLC11A2 | solute carrier family 11 (proton-coupled divalent metal ion transporters), member 2 | 1.37 | 6.99035E-05 | 210047_at |
| MEIS2 | Meis1, myeloid ecotropic viral integration site 1 homolog 2 (mouse) | 0.28 | 7.10855E-05 | 1563841_at |
| PLA2G12B | phospholipase A2, group XIIB /// phospholipase A2, group XIIB | 1.57 | 7.25546E-05 | 224411_at |
| CYB5-M | outer mitochondrial membrane cytochrome b5 | 2.03 | 7.55266E-05 | 201633_s_at |
| HSD17B12 | hydroxysteroid (17-beta) dehydrogenase 12 | 1.85 | 7.55268E-05 | 1554122_a_at |
| UROD | uroporphyrinogen decarboxylase | 0.72 | 7.59078E-05 | 208970_s_at |
| KBTBD2 | kelch repeat and BTB (POZ) domain containing 2 | 1.19 | 7.63403E-05 | 234232_at |
| BBX | Bobby sox homolog (Drosophila) | 0.40 | 7.7266E-05 | 1557239_at |
| DPY19L2 | dpy-19-like 2 (C. elegans) | 0.62 | 7.73583E-05 | 230158_at |
| MAPKAPK5 | mitogen-activated protein kinase-activated protein kinase 5 | 1.85 | 7.74363E-05 | 212871_at |
| SNX27 | sorting nexin family member 27 /// sorting nexin family member 27 | 0.77 | 7.85111E-05 | 221006_s_at |
| CDX2 | caudal type homeo box transcription factor 2 | 1.86 | 7.93668E-05 | 206387_at |
| LOC90268 | hypothetical protein BC007706 | 1.21 | 7.99505E-05 | 240834_at |
| EIF4E2 | Eukaryotic translation initiation factor 4E member 2 | 0.87 | 8.1256E-05 | 244302_at |
| CKAP2 | cytoskeleton associated protein 2 | 2.53 | 8.15552E-05 | 218252_at |
| --- | MRNA; cDNA DKFZp686E16168 (from clone DKFZp686E16168) | 1.69 | 8.29499E-05 | 238565_at |
| RNF19 | Ring finger protein 19 | 0.51 | 8.31156E-05 | 230599_at |
| AKAP13 | A kinase (PRKA) anchor protein 13 | 0.54 | 8.33452E-05 | 224884_at |
| CDCA2 | cell division cycle associated 2 | 1.54 | 8.36786E-05 | 236957_at |
| GOT2 | glutamic-oxaloacetic transaminase 2, mitochondrial (aspartate aminotransferase 2) | 1.50 | 8.42792E-05 | 200708_at |
| PHF19 | PHD finger protein 19 | 1.93 | 8.50867E-05 | 227212_s_at |
| HDCMA18P | HDCMA18P protein | 0.73 | 8.74534E-05 | 241385_at |
| LRRC28 | leucine rich repeat containing 28 | 1.31 | 8.83436E-05 | 227423_at |
| --- | --- | 1.39 | 8.83449E-05 | 216532_x_at |
| --- | Transcribed locus | 1.66 | 8.86317E-05 | 235363_at |
| PHF21A | PHD finger protein 21A | 0.67 | 8.86982E-05 | 203278_s_at |
| FAM29A | family with sequence similarity 29, member A | 1.51 | 8.89525E-05 | 233655_s_at |
| LOC63928 | hepatocellular carcinoma antigen gene 520 | 3.56 | 8.9231E-05 | 206149_at |
| FLJ20464 | hypothetical protein FLJ20464 | 1.20 | 8.9599E-05 | 224073_at |
| SEC23B | Sec23 homolog B (S. cerevisiae) | 2.03 | 9.16391E-05 | 210293_s_at |
| MYST3 | MYST histone acetyltransferase (monocytic leukemia) 3 | 0.58 | 9.30965E-05 | 242480_at |
| SVIL | Supervillin | 0.75 | 9.4045E-05 | 215279_at |
| SLC11A2 | solute carrier family 11 (proton-coupled divalent metal ion transporters), member 2 | 2.72 | 9.40876E-05 | 203123_s_at |
| APOL2 | apolipoprotein L, 2 /// apolipoprotein L, 2 | 0.78 | 9.43019E-05 | 221013_s_at |
| HGF | hepatocyte growth factor (hepapoietin A; scatter factor) | 1.29 | 9.49287E-05 | 210998_s_at |
| GPA33 | glycoprotein A33 (transmembrane) | 5.07 | 9.50323E-05 | 205929_at |
| KIF2C | kinesin family member 2C | 1.69 | 9.56756E-05 | 211519_s_at |
| MLPH | Melanophilin | 0.32 | 9.60639E-05 | 229150_at |
| YPEL2 | Yippee-like 2 (Drosophila) | 0.67 | 9.61399E-05 | 244200_at |
| --- | --- | 1.58 | 9.75867E-05 | 238228_at |
| ABCD3 | ATP-binding cassette, sub-family D (ALD), member 3 | 1.65 | 9.78152E-05 | 1554878_a_at |
| LOC143458 | Low density lipoprotein receptor class A domain containing 3 | 0.76 | 9.85161E-05 | 1557889_at |
| NOTCH2NL | Notch homolog 2 (Drosophila) N-terminal like | 0.42 | 9.90765E-05 | 214722_at |
| KIAA0495 | KIAA0495 | 0.44 | 9.91858E-05 | 213340_s_at |
| MYEF2 | myelin expression factor 2 | 0.19 | 9.9977E-05 | 222771_s_at |
| INPP4A | Inositol polyphosphate-4-phosphatase, type I, 107kDa | 0.61 | 0.000100274 | 235695_at |
| CKLFSF7 | chemokine-like factor superfamily 7 | 0.56 | 0.000100608 | 226017_at |
| DMPK | dystrophia myotonica-protein kinase | 0.75 | 0.000101586 | 217066_s_at |
| ACOT7 | acyl-CoA thioesterase 7 | 2.28 | 0.000101857 | 208002_s_at |
| --- | --- | 0.35 | 0.000104375 | 231136_at |
| C4orf9 | Chromosome 4 open reading frame 9 | 1.11 | 0.000105664 | 215746_at |
| ACCN1 | amiloride-sensitive cation channel 1, neuronal (degenerin) | 1.35 | 0.000105779 | 206690_at |
| LOC51066 | fls485 | 1.94 | 0.000108047 | 206741_at |
| FSHPRH1 | FSH primary response (LRPR1 homolog, rat) 1 | 1.45 | 0.000108251 | 207590_s_at |
| DEPDC1 | DEP domain containing 1 | 2.02 | 0.000108889 | 232278_s_at |
| GNA11 | guanine nucleotide binding protein (G protein), alpha 11 (Gq class) | 1.58 | 0.000109657 | 204248_at |
| DIAPH3 | Diaphanous homolog 3 (Drosophila) | 1.88 | 0.000111695 | 229097_at |
| --- | LOC440309 | 0.50 | 0.000113836 | 244443_at |
| MAST4 | microtubule associated serine/threonine kinase family member 4 | 0.53 | 0.000115533 | 40016_g_at |
| MCM7 | MCM7 minichromosome maintenance deficient 7 (S. cerevisiae) | 2.72 | 0.000118321 | 210983_s_at |
| FLJ36874 | hypothetical protein FLJ36874 | 1.43 | 0.000120713 | 225466_at |
| AP1S1 | adaptor-related protein complex 1, sigma 1 subunit | 1.22 | 0.000120828 | 205196_s_at |
| EPHA1 | EPH receptor A1 | 1.92 | 0.000121297 | 205977_s_at |
| --- | CDNA FLJ35260 fis, clone PROST2004258 | 0.29 | 0.000122115 | 227997_at |
| SSBP2 | Single-stranded DNA binding protein 2 | 0.56 | 0.000123395 | 1557813_at |
| KIF18A | kinesin family member 18A /// kinesin family member 18A | 1.94 | 0.000125348 | 221258_s_at |
| --- | CDNA FLJ14188 fis, clone NT2RP2005980 | 0.82 | 0.000125611 | 233354_at |
| ZDHHC2 | Zinc finger, DHHC-type containing 2 | 0.42 | 0.00012617 | 243528_at |
| MKI67 | antigen identified by monoclonal antibody Ki-67 | 2.55 | 0.000126652 | 212021_s_at |
| TOPORS | Topoisomerase I binding, arginine/serine-rich | 1.18 | 0.000127639 | 221979_at |
| TPM2 | tropomyosin 2 (beta) | 0.17 | 0.000127924 | 204083_s_at |
| CDKN3 | cyclin-dependent kinase inhibitor 3 (CDK2-associated dual specificity phosphatase) | 2.80 | 0.000128391 | 209714_s_at |
| SPHK2 | sphingosine kinase 2 | 1.54 | 0.000128994 | 40273_at |
| CDCA5 | cell division cycle associated 5 | 2.07 | 0.000129043 | 224753_at |
| PX19 | px19-like protein | 1.73 | 0.000129063 | 223032_x_at |
| CHEK1 | CHK1 checkpoint homolog (S. pombe) | 4.20 | 0.000129613 | 205394_at |
| CLU | clusterin (complement lysis inhibitor, SP-40,40, sulfated glycoprotein 2, testosterone-repressed prostate message 2, apolipoprotein J) | 0.12 | 0.000130733 | 222043_at |
| ETHE1 | ethylmalonic encephalopathy 1 | 2.73 | 0.000132845 | 204034_at |
| KIAA0152 | KIAA0152 | 1.80 | 0.000133451 | 200617_at |
| CENPH | centromere protein H | 1.97 | 0.000133762 | 231772_x_at |
| NSE1 | Family with sequence similarity 84, member A | 1.65 | 0.000134134 | 234335_s_at |
