# Supplementary material for: Gene Expression Profiling of Ampullary Carcinomas Classifies Ampullary Carcinomas into Biliary-Like and Intestinal-Like Subtypes That Are Prognostic of Outcome
Source: PLoS One. 2013 Jun 11;8(6):e65144. doi: 10.1371/journal.pone.0065144 (PMC3679143; doi:10.1371/journal.pone.0065144)
Supplement: Table S2 — Quantitative protein expression data between intestinal-like and biliary-like ampullary subtypes. (DOCX) [file pone.0065144.s004.docx]

Table S2: Quantitative protein expression data between intestinal-like and biliary-like ampullary subtypes

| Antibody | Fold Change | P-value | Antibody | Fold Change | P-value |
| --- | --- | --- | --- | --- | --- |
| MAPK_pT202 | 0.32 | <0.001 | m-TOR | 0.820 | 0.265 |
| Stat3_pS727 | 0.87 | <0.001 | p38/MAPK | 1.138 | 0.270 |
| Rad51 | 1.39 | 0.001 | PI3Kp110a | 0.890 | 0.276 |
| Cyclin B1 | 3.27 | 0.002 | PR | 0.905 | 0.276 |
| Raf-B | 0.74 | 0.002 | NFkBp64_pS536 | 1.329 | 0.278 |
| GSK3_pS21 | 0.71 | 0.002 | Notch3 | 0.852 | 0.284 |
| PCNA | 1.44 | 0.003 | p21 | 0.714 | 0.295 |
| Chk1 | 1.17 | 0.003 | K-RAS | 1.083 | 0.297 |
| Src_pY527 | 0.68 | 0.005 | Rb | 1.213 | 0.299 |
| mTOR_pS2448 | 0.71 | 0.005 | Beclin | 0.941 | 0.306 |
| Raf-C_pS338 | 0.73 | 0.006 | Paxillin | 0.891 | 0.330 |
| Raf-A_pS299 | 0.77 | 0.007 | Annexin | 0.766 | 0.331 |
| Collagen VI | 0.31 | 0.009 | BIM | 1.206 | 0.333 |
| YB1 | 1.27 | 0.011 | Taz | 0.937 | 0.335 |
| PKCa | 0.63 | 0.012 | IRS-1 | 0.901 | 0.338 |
| 4EBP1 | 1.48 | 0.014 | MEK1 | 0.902 | 0.350 |
| IGFR1b | 0.74 | 0.014 | YAP | 1.065 | 0.356 |
| c-Myc_pT58 | 1.42 | 0.014 | Src | 0.948 | 0.358 |
| FOXO3a_pS318 | 0.35 | 0.015 | INPP4B | 0.867 | 0.358 |
| AMPK_pT172 | 1.71 | 0.015 | S6 | 1.184 | 0.358 |
| Cadherin-E | 1.64 | 0.017 | Src_pY416 | 1.723 | 0.370 |
| Caspase 3 Active | 1.16 | 0.017 | p70S6K | 0.967 | 0.379 |
| NF2 | 0.82 | 0.018 | PARP cleaved | 1.464 | 0.382 |
| Transglutaminase | 0.74 | 0.018 | AKT | 0.921 | 0.391 |
| Myc | 1.51 | 0.018 | SOCS2 | 0.941 | 0.398 |
| Caspase 7 cleaved Asp198 | 2.49 | 0.022 | DJ-1 | 1.246 | 0.404 |
| Bcl-X | 1.24 | 0.024 | p53 | 1.057 | 0.420 |
| p70S6K_pT389 | 0.86 | 0.024 | p90RSK_pT359 | 0.926 | 0.421 |
| AKT_pS473 | 0.59 | 0.026 | AMPK | 0.921 | 0.427 |
| PKC_pS657 | 0.69 | 0.030 | Raf-C | 0.911 | 0.435 |
| YAP_pS127 | 0.72 | 0.032 | AIB-1 | 1.121 | 0.449 |
| Bid | 1.25 | 0.035 | 53BP1 | 0.912 | 0.463 |
| S6_pS240 | 1.46 | 0.036 | Rb_pS807 | 1.582 | 0.477 |
| Fibronectin | 0.44 | 0.037 | Cyclin D1 | 1.062 | 0.494 |
| MEK1_pS217 | 0.75 | 0.038 | p27 | 0.912 | 0.497 |
| MEK1-2_pS217 | 0.75 | 0.041 | HER2_pY1248 | 1.025 | 0.504 |
| Pea15 | 0.84 | 0.052 | HER2 | 0.842 | 0.505 |
| Catenin Beta | 1.70 | 0.052 | 4EBP1_pS65 | 0.928 | 0.518 |
| Jnk2 | 0.83 | 0.056 | XRCC1 | 1.036 | 0.540 |
| BAX | 1.66 | 0.057 | Cyclin E1 | 1.057 | 0.549 |
| VEGFR2 | 0.53 | 0.063 | Tuberin/TSC2 | 0.884 | 0.549 |
| eIF4E | 1.16 | 0.063 | EGFR_pY1173 | 0.966 | 0.552 |
| Chk2_pT68 | 1.20 | 0.066 | PTEN | 0.900 | 0.555 |
| Vasp | 1.19 | 0.067 | PI3Kp85 | 0.953 | 0.560 |
| ACC_pS79 | 1.66 | 0.076 | KU80 | 1.199 | 0.569 |
| Smead3 | 0.78 | 0.083 | Stat5_pY694 | 1.118 | 0.573 |
| EGFR | 0.91 | 0.085 | Xiap | 1.049 | 0.599 |
| AR | 0.83 | 0.086 | MSH2 | 0.862 | 0.605 |
| Claudin 7 | 1.87 | 0.090 | Smad4 | 0.891 | 0.620 |
| Stat5 | 0.78 | 0.090 | p38_pT180 | 0.878 | 0.637 |
| Pea15_pS116 | 1.60 | 0.097 | PDK1_pS241 | 1.116 | 0.638 |
| GSK3-Beta | 0.86 | 0.105 | cJun_pS73 | 0.950 | 0.641 |
| Tau | 0.93 | 0.109 | Bcl-2 | 1.128 | 0.645 |
| Bcl-Xl | 1.10 | 0.111 | FOXO3a | 1.083 | 0.657 |
| Chk2 | 0.71 | 0.113 | Cadherin-N | 0.971 | 0.677 |
| ER-a_pS118 | 0.93 | 0.121 | CDC2 | 1.023 | 0.685 |
| Chk1_pS345 | 1.06 | 0.130 | p53 | 0.973 | 0.699 |
| ER | 0.88 | 0.140 | ACC1 | 1.180 | 0.720 |
| Shc_pY317 | 0.88 | 0.144 | Stat3_pS705 | 1.090 | 0.729 |
| Kit-C | 0.48 | 0.148 | Bak | 0.976 | 0.733 |
| p27_pT198 | 1.57 | 0.151 | Mre11 | 0.981 | 0.764 |
| Rad50 | 0.92 | 0.171 | Taz_pS89 | 1.028 | 0.769 |
| Rab25 | 0.80 | 0.177 | Caveolin 1 | 0.901 | 0.855 |
| FAK | 1.83 | 0.188 | CD31 | 1.002 | 0.882 |
| HIF1 | 1.33 | 0.204 | IGFBP2 | 1.018 | 0.903 |
| Gata3 | 0.85 | 0.215 | Cox-2 | 1.010 | 0.913 |
| EGFR_pY992 | 0.92 | 0.244 | Stathmin | 1.016 | 0.918 |
| YB1_pS102 | 1.24 | 0.256 | Snail | 0.610 | 0.918 |
| AKT_pT308 | 0.80 | 0.264 | Cadherin-P | 0.962 | 0.959 |
| Pras40_pT246 | 1.07 | 0.264 | S6_pS235 | 0.823 | 0.996 |
