# Supplementary material for: Gene Expression Profiling of Ampullary Carcinomas Classifies Ampullary Carcinomas into Biliary-Like and Intestinal-Like Subtypes That Are Prognostic of Outcome
Source: PLoS One. 2013 Jun 11;8(6):e65144. doi: 10.1371/journal.pone.0065144 (PMC3679143; doi:10.1371/journal.pone.0065144)
Supplement: Methods S1 — Methodology for tissue microarray construction, immunohistochemical analysis, microsatellite instability determination, and DNA mutation analysis. (DOCX) [file pone.0065144.s005.docx]

**Tissue Microarray Construction**

The formalin-fixed, paraffin-embedded archival tissue blocks and their matching hematoxylin and eosin-stained slides were retrieved, reviewed and screened for representative tumor regions. Three cores of tumor tissue were sampled from representative areas using a 1.0-mm punch. The tissue microarray was constructed using a tissue microarrayer (Beecher Instruments, Sun Prairie, WI) as described previously (Wang H, Wang H, Zhang W, et al: Tissue microarrays: applications in neuropathology research, diagnosis, and education. Brain Pathol 12:95-107, 2002). The constructed TMA blocks were sealed with paraffin, and 5-µm-thick slides were cut from the TMA blocks for immunohistochemical staining of CDX-2, CK7, and CK20. Expression for each marker was considered positive if 10% or more of the tumor cells across the three cores demonstrated staining as determined by a gastrointestinal pathologist (HW).

**Immunohistochemical Analysis**

Immunohistochemical staining was conducted using anti-CDX-2 (CDX-88, 1:50 dilution, Biogenex, San Ramon, CA), anti-CK7 (OVT-TL12/30, 1:100 dilution, Dako, Carpinteria, CA), anti-CK20 (KS20.8, 1:4000 dilution, Dako, Carpinteria, CA), anti-PMS2 (A16-4, 1:125 dilution, B.D. Biosciences, San Jose, CA), anti-MSH-6 (44, 1:300 dilution, B.D. Biosciences, San Jose, CA), anti-MSH-2 (FE11, 1:100 dilution, Calbiochem Inc,, Gibbstown, NJ), anti-MLH1 (G168-15, 1:25 dilution, B.D. Biosciences, San Jose, CA). To retrieve the antigenicity, the tissue sections were treated at 100 ºC in a steamer containing 10 mmol citrate buffer (pH, 6.0) for 60 min. The sections were then immersed in methanol containing 0.3% hydrogen peroxidase for 20 min to block the endogenous peroxidase activity and were incubated in 2.5% blocking serum to reduce nonspecific binding. Sections were incubated for 90 min at 37°C with primary antibodies at the dilutions specified. Standard avidin-biotin immunohistochemical analysis of the sections was done according to the manufacturer’s recommendations (Vector Laboratories, Burlingame, CA). Diaminobenzidine tetrahydrochloride was used as a chromogen, and hematoxylin was used for counterstaining.

**Microsatellite Instability Determination by PCR Analysis**

Tumor DNA was isolated from each frozen sample using QIAmp DNA Mini Kit (Qiagen Inc., Valencia, CA). For normal control, representative slides of normal small bowel mucosa were obtained for each case and macro-dissection of the relevant tissue was done. DNA was extracted by proteinase K digestion. Genomic DNA was amplified by polymerase chain reaction (PCR) utilizing fluorescently labeled and unlabeled primers for each of five National Cancer Institute–recommended microsatellite markers (BAT25, BAT26, D2S123, D5S346, and D17S250). PCR products were electrophoretically separated using an ABI 3130 analyzer (Applied Biosystems, Foster City, CA, USA) and analyzed by GeneMapper 4.0 software (Applied Biosystems). For two samples, in which normal tissue was not available, immunohistochemical analysis of mismatch repair proteins, including MLH1, MSH2, MSH6 and PMS2, was used to determine MSI status. Lost nuclear expression in comparison to respective normal small bowel of any of the four mismatch repair proteins was considered MSI-high.

**DNA Mutation Analysis**

The Sequenom^®^ high-throughput MassARRAY platform was used to detect single nucleotide polymorphisms as previously described.[[21](#_ENREF_21)] The following mutations in KRAS (KRAS_G12A, KRAS_G12C, KRAS_G12D, KRAS_G13A, KRAS_G13V, KRAS_Q61H, KRAS_Q61H), BRAF (BRAF_V600D, BRAF_V600E, BRAF_V600K), and PIK3CA (PIK3CA_A1046V, PIK3CA_C420R, PIK3CA_E110K, PIK3CA_E418K, PIK3CA_E453K, PIK3CA_E542K, PIK3CA_E542V, PIK3CA_E545A, PIK3CA_E545K, PIK3CA_F909L, PIK3CA_G1049R, PIK3CA_H1047L, PIK3CA_H1047R, PIK3CA_H1047Y, PIK3CA_H701P, PIK3CA_K111N, PIK3CA_M1043V, PIK3CA_N345K, PIK3CA_P539R, PIK3CA_Q060K, PIK3CA_Q546E, PIK3CA_Q546K, PIK3CA_Q546R, PIK3CA_R088Q, PIK3CA_S405F, PIK3CA_S405F, PIK3CA_T1025S) were evaluated. Duplicate Spectrochip matrix chips were run and reactions with >15% of the resultant mass ran in the mutant site in both reactions were scored as positive.
